# Spatially continuous dataset at local scale of Taita Hills in Kenya and Mount Kilimanjaro in Tanzania

**DOI:** 10.1016/j.dib.2016.07.041

**Published:** 2016-07-26

**Authors:** Sizah Mwalusepo, Estomih S. Massawe, Tino Johansson

**Affiliations:** a icipe—African Insect Science for Food and Health, P.O. Box 30772-00100, Nairobi, Kenya; b Department of General Studies, Dar es Salaam Institute of Technology, P.O. Box 2958, Dar es Salaam, Tanzania; c Department of M athematics, University of Dar es Salaam, P.O. Box 35062, Dar es Salaam, Tanzania; d Department of Geosciences and Geography, University of Helsinki, P.O. Box 68, FI-00014, Finland

**Keywords:** Spatial climate data, Climate change, Modeling, Local scale

## Abstract

Climate change is a global concern, requiring local scale spatially continuous dataset and modeling of meteorological variables. This dataset article provided the interpolated temperature, rainfall and relative humidity dataset at local scale along Taita Hills and Mount Kilimanjaro altitudinal gradients in Kenya and Tanzania, respectively. The temperature and relative humidity were recorded hourly using automatic onset ^TH^HOBO data loggers and rainfall was recorded daily using GENERAL^R^ wireless rain gauges. Thin plate spline (TPS) was used to interpolate, with the degree of data smoothing determined by minimizing the generalized cross validation. The dataset provide information on the status of the current climatic conditions along the two mountainous altitudinal gradients in Kenya and Tanzania. The dataset will, thus, enhance future research.

**Specifications Table**

**Table:** 

Subject area	Agriculture, environmental science and climate change
More specific subject area	Spatial continuous dataset
Type of data	Figures
How data was acquired	Thin Plate spline algorithm was fitted; with Computer program written in R software
Data format	Interpolated, analyzed
Experimental factors	Automatic onset ^TH^HOBO data loggers and General ^R^ wireless rain gauges were installed at different altitude to track temperatures, Rainfall and relative humidity.
Experimental features	Thin plate spline (TPS) interpolation was carried out using R software link with GIS software (ArcGIS version 10.1) to generate interpolation maps showing the spatially continuous data over the study areas.
Data source location	Taita Hills, Kenya; Mount Kilimanjaro, Tanzania.
Data accessibility	Data are available in this article

**Value of the data**

• The data provide information on the status of the climatic conditions along the two mountain altitudinal gradients in Kenya and Tanzania, and includes data accessible for reuse.
• The data are important to farming communities, agricultural extension agents, government institutions, international and local non-governmental organizations engaged in agricultural interventions, including useful data to researchers, students and academics.
• The data can be used for mapping and spatial modeling in a geographical information system (GIS).
• The data is valuable for improvements computational facilities, insufficient climate data and reliable downscaling at a local scale.
• The data are important in making confidentially informed decisions, giving scientists accurate spatially continuous data cross a region for making justified interpolation.

## 1 Data

**Fig. 1:**
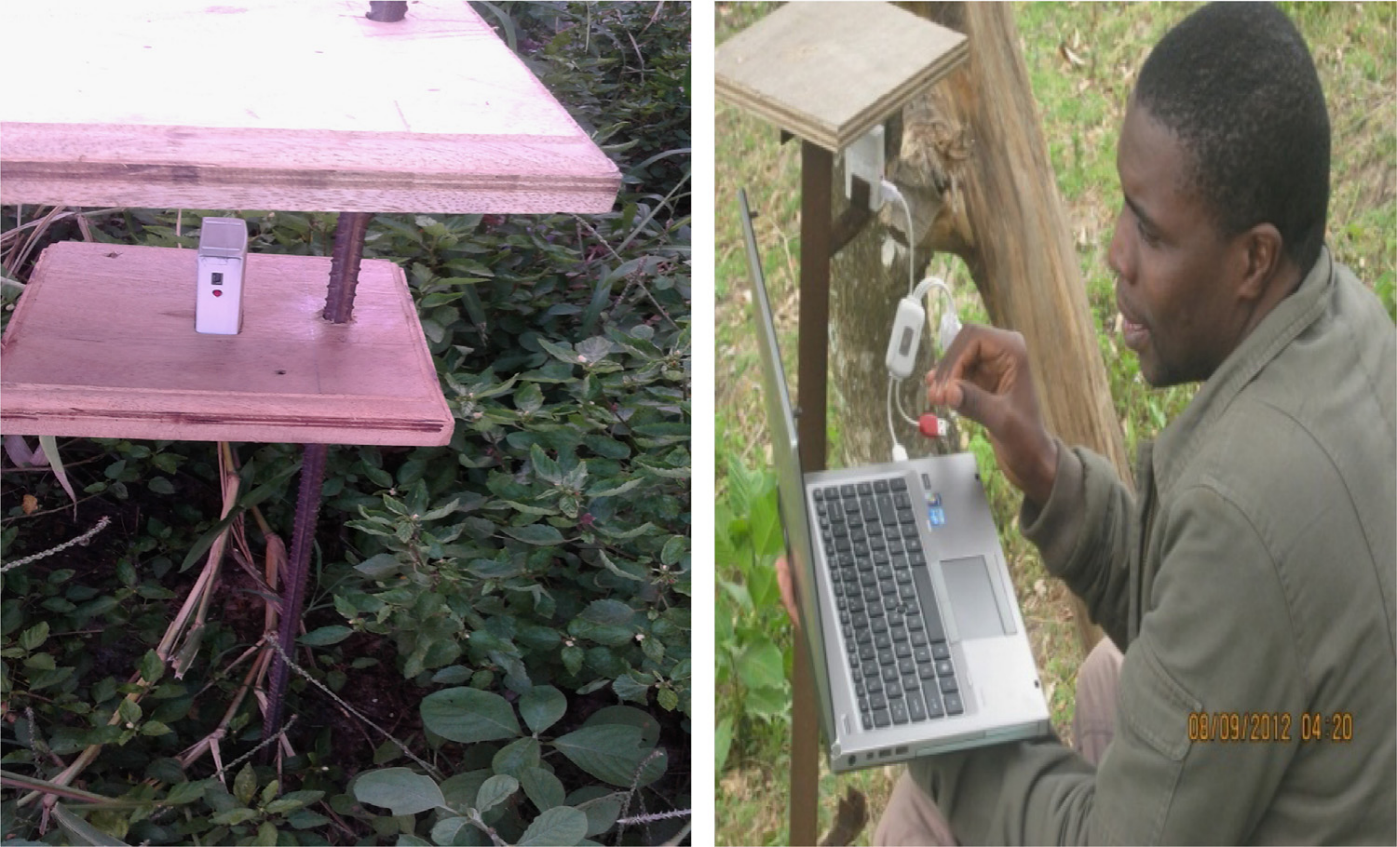
Data loggers.

**Fig. 2:**
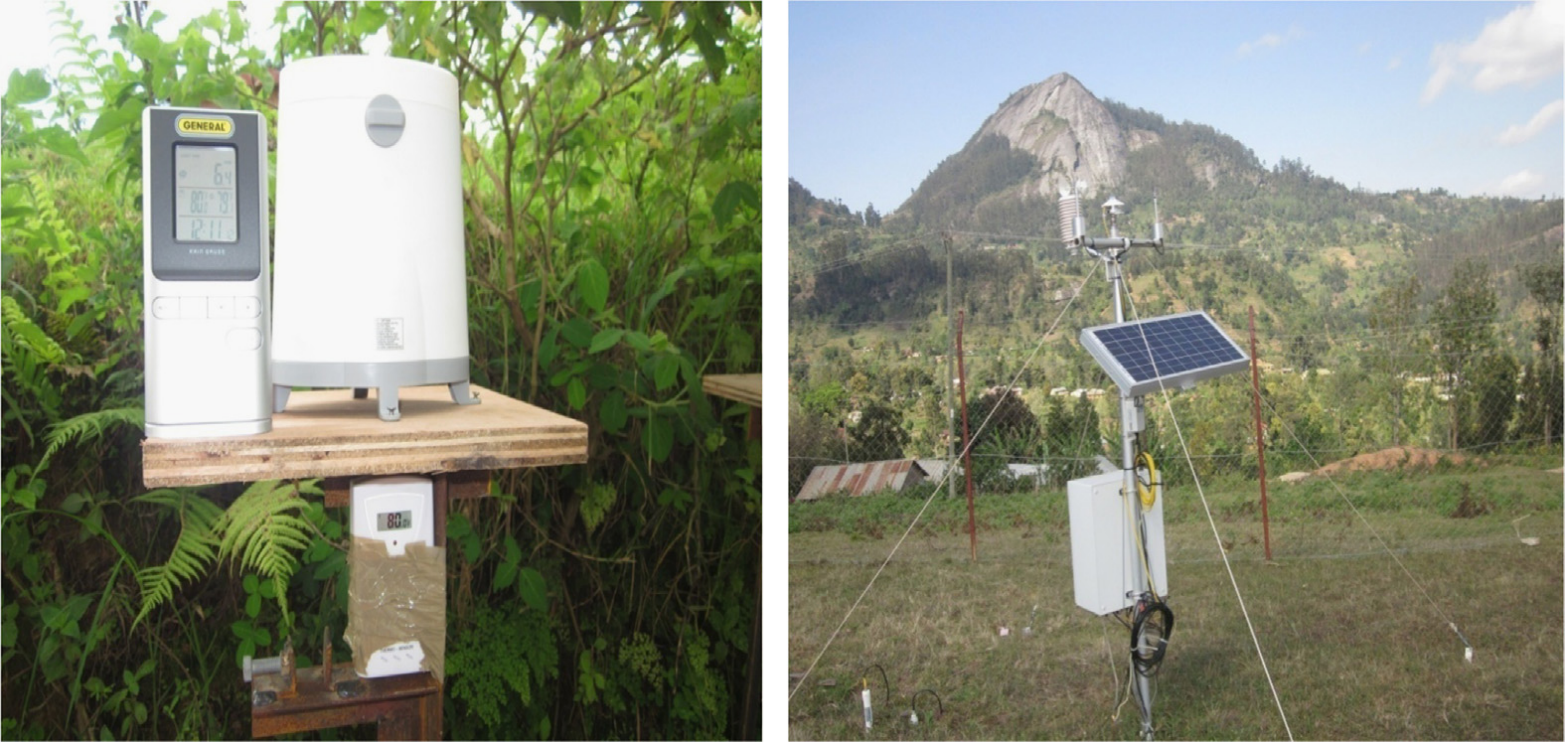
Raingauges.

**Fig. 3:**
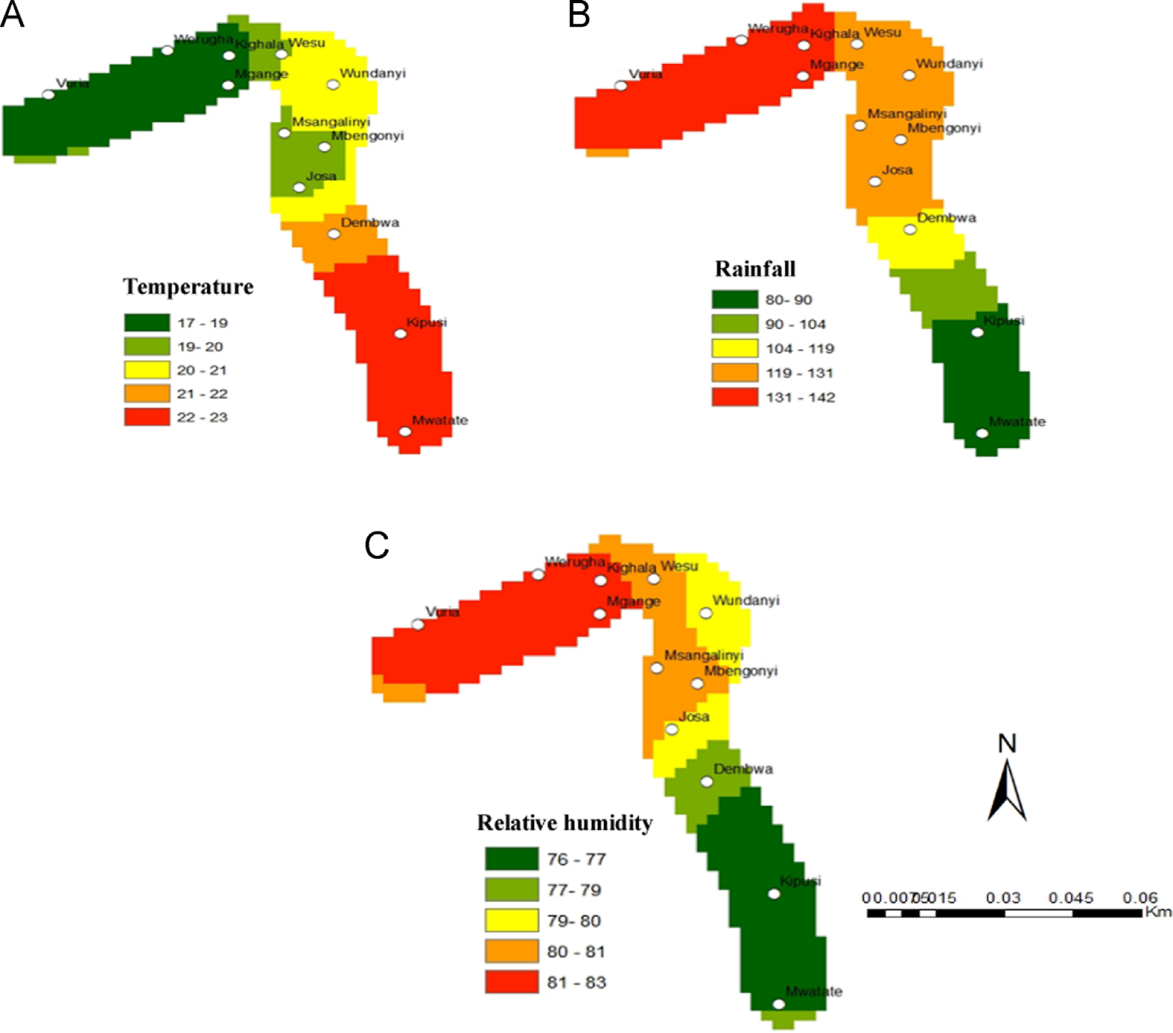
Mean annual temperature (A); annual rainfall (B); relative humidity (C) along Taita Hills.

**Fig. 4:**
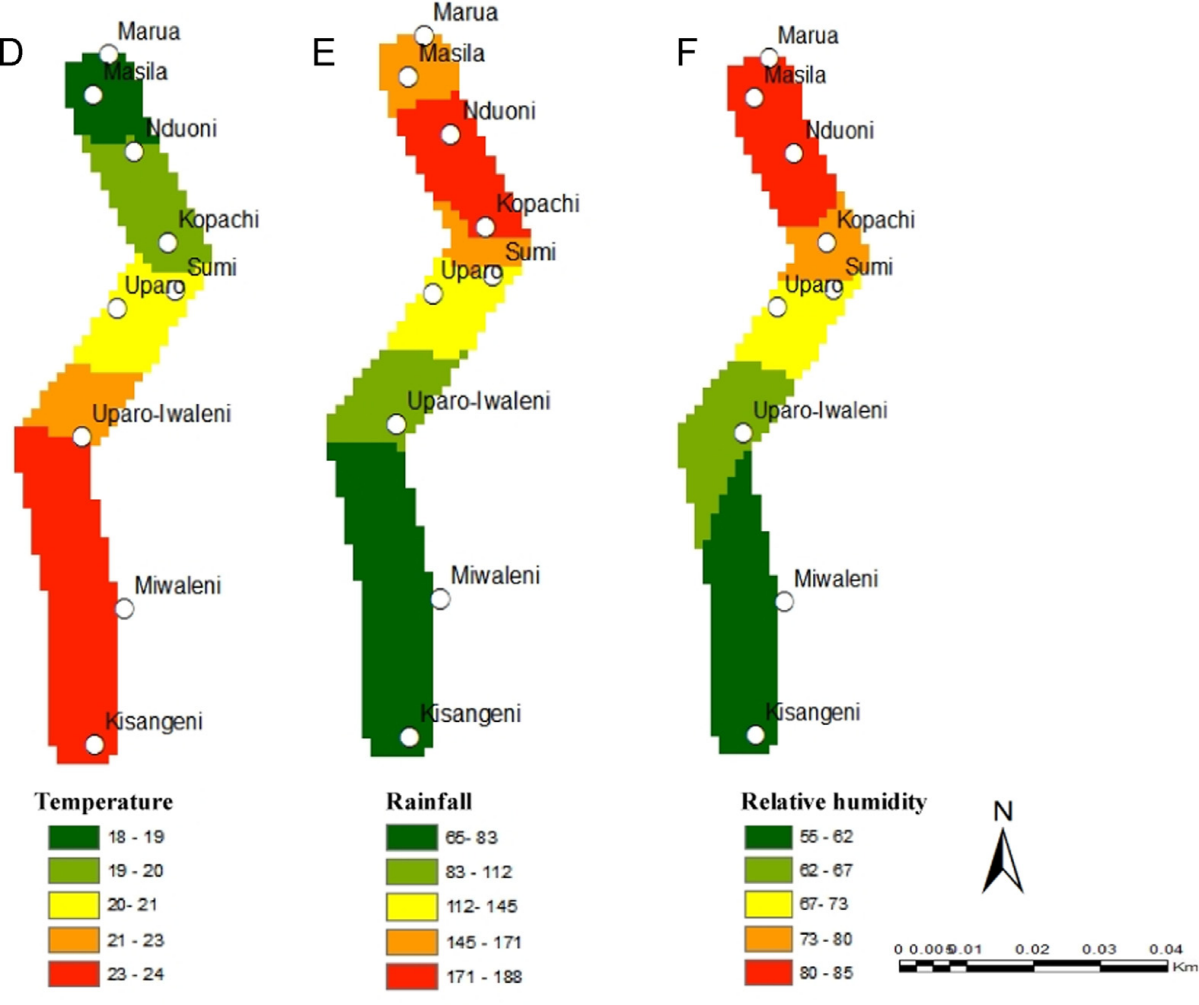
Mean annual temperature (D); annual rainfall (E); relative humidity (F) along Mount Kilimanjaro.

Fig. 1, Fig. 2 show the data on sample collection and how the data loggers and rain gauges have been installed along the altitudinal gradients. Fig. 3 presents spatially continuous data for temperatures (A); rainfall (B); and relative humidity (C) along Taita Hills. Fig. 4 presents spatially continuous data for temperatures (D); rainfall (E); and relative humidity (F) along Mount Kilimanjaro under current climatic condition.

## 2 Experimental design, materials and methods

The details of the sites have been described in our previous study [1]. In brief, the dataset collections are localized in Kenya and Tanzania. In Kenya, was situated in Taita Hills in South-Eastern Kenya (coastal region), between latitude 3°25′ and longitude 38°20′. In Tanzania, was situated in the Pangani river basin in North East (NE) Tanzania with a focus on the small catchment areas on the South Eastern slope of Mount Kilimanjaro approximately located between latitude 3°4′ and longitude 37°4′. The temperature, relative humidity and rainfall required for carrying out the spatial interpolation were obtained from local weather stations. Automatic onset ™HOBO data loggers (hourly records) and GENERAL^R^ wireless rain gauges were installed at station across study sites to keep track of daily temperatures, relative humidity and rainfall, respectively [1], [2]. The *x*–*y* coordinates position and altitudes were recorded using a Global Positioning System (GPS) (German eTrex Vista^(R)^). The thin plate spline (TPS) algorithm [3] was used to interpolate temperature, rainfall and relative humidity dataset. Data processing and analysis were carried out with a computer program written in R software [4] and linked with Geographic Information System (GIS). The accuracy of the environmental variables surface was assessed by comparing surface values withheld from the interpolation procedure. Three statistical criteria were used to evaluate accuracy namely: (i) R-square (R^2^); (ii) the Root Mean Square Error (RMSE); and (iii) the Relative Root Mean Square Error (RMSEr).
