# Supplementary material for: Spatially continuous dataset at local scale of Taita Hills in Kenya and Mount Kilimanjaro in Tanzania
Source: Data Brief. 2016 Jul 26;8:1115–9. doi: 10.1016/j.dib.2016.07.041 (PMC4976666; doi:10.1016/j.dib.2016.07.041)
Supplement: Supplementary file 1 — Supplementary material [file mmc1.doc]

Editorial Board for Journal of Data in Brief

Manuscript Draft

Manuscript Number:

Title: **Spatially continuous dataset at local scale of Taita Hills in Kenya and Mount Kilimanjaro in Tanzania**

Article Type: Data Article

**Keywords**: Spatial climate data; climate change; modeling; local scale

**Corresponding Author**: Sizah Mwalusepo

***E-mail address*:** [mwalusepo@yahoo.com](mailto:mwalusepo@yahoo.com)/[smwalusepo@icipe.org](mailto:smwalusepo@icipe.org)

***Mobile phone***: +254700459981/+255754864493

**First Author**: Sizah Mwalusepo

**Order of Authors**: Sizah Mwalusepo, Estomih S. Massawe, Tino Johansson

**Abstract**: Climate change is a global concern, requiring local scale spatially continuous dataset and modeling of meteorological variables. This dataset article provided the interpolated temperature, rainfall and relative humidity dataset at local scale along Taita Hills and Mount Kilimanjaro altitudinal gradients in Kenya and Tanzania, respectively. The temperature and relative humidity were recorded hourly using automatic onset^TH^HOBO data loggers and rainfall was recorded daily using GENERAL^R^ wireless rain gauges. Thin plate spline (TPS) was used to interpolate, with the degree of data smoothing determined by minimizing the generalized cross validation. The dataset provide information on the status of the current climatic conditions along the two mountainous altitudinal gradients in Kenya and Tanzania. The dataset will, thus enhance future research.

**Conflicts of interest**

July 11, 2016.

Editor-in-Chief

Dear Editor:

Enclosed for your consideration is an original research article, entitled “**Spatially continuous dataset at local scale of Taita Hills in Kenya and Mount Kilimanjaro in Tanzania”,** which we submit for publication in the Journal of Data in Brief. Here, I declare that:

1. All authors of this research paper have directly participated in the planning, execution, or analysis of this study.
2. The contents of this manuscript have not been copyrighted or published previously.
3. The contents of this manuscript are not now under consideration for publication elsewhere.
4. The contents of this manuscript will not be copyrighted, submitted, or published elsewhere, while acceptance by the Journal is under consideration.
5. There are no directly related manuscripts, published or unpublished, by any authors of this paper.
6. The paper contain only four figures

We think this work bring a valuable contributions to the understanding spatial continuous dataset at a local scale in Kenya and Tanzania. Hence we hope that these interesting dataset make of this study candidate of interest for your journal.

Sincerely yours,

Sizah Mwalusepo (on behalf of all co-authors)
